# The impact of the interferon-lambda family on the innate and adaptive immune response to viral infections

**DOI:** 10.1038/emi.2014.51

**Published:** 2014-07-16

**Authors:** Adrian Egli, Deanna M Santer, Daire O'Shea, D Lorne Tyrrell, Michael Houghton

**Affiliations:** 1 Infection Biology, Department of Biomedicine, University Hospital of Basel, 4031 Basel, Switzerland; 2 Clinical Microbiology, University Hospital of Basel, 4031 Basel, Switzerland; 3 Department of Medical Microbiology and Immunology, and Li Ka Shing Institute of Virology, University of Alberta, Edmonton, Alberta T6G 2E1, Canada; 4 Division of Infectious Diseases, University of Alberta, Edmonton, Alberta T6G 2E1, Canada

**Keywords:** B cells, hepatitis C virus, immune response, innate immunity, interferon-lambda, receptor, respiratory viruses, T cells

## Abstract

Type-III interferons (IFN-λ, IFNL) are the most recently described family of IFNs. This family of innate cytokines are increasingly being ascribed pivotal roles in host–pathogen interactions. Herein, we will review the accumulating evidence detailing the immune biology of IFNL during viral infection, and the implications of this novel information on means to advance the development of therapies and vaccines against existing and emerging pathogens. IFNLs exert antiviral effects via induction of IFN-stimulated genes. Common single nucleotide polymorphisms (SNPs) in the IFNL3, IFNL4 and the IFNL receptor α-subunit genes have been strongly associated with IFN-α-based treatment of chronic hepatitis C virus infection. The clinical impact of these SNPs may be dependent on the status of viral infection (acute or chronic) and the potential to develop viral resistance. Another important function of IFNLs is macrophage and dendritic cell polarization, which prime helper T-cell activation and proliferation. It has been demonstrated that IFNL increase Th1- and reduce Th2-cytokines. Therefore, can such SNPs affect the IFNL signaling and thereby modulate the Th1/Th2 balance during infection? In turn, this may influence the subsequent priming of cytotoxic T cells versus antibody-secreting B cells, with implications for the breadth and durability of the host response.

## GENERAL ASPECTS OF INTERFERONS (IFNS)

Since their inception, IFNs have displayed ever increasing diversity and demonstrated activity far more refined than the original observation of the ability to ‘interfere' with viral replication.^1^ Each IFN family member mediates important anti-viral activity via engagement with their respective IFN receptor. All IFNs share an α-helical bundle structure and broadly function in a similar manner however significant effector differences exist.^2^ IFNs are divided into three types: type-I mainly represented by IFN-α^3,4,5^ and -β,^6,7^ type-II by IFN-γ,^8^ and type III consulting the recently discovered IFN-lambda (IFNL) family.^9,10,11^ We propose that IFNL occupies a key position modulating the host response to pathogens. Comprehensive understanding of the immunological impact may become increasingly important to understand host-virus interactions of existing and emerging pathogens, with application to the development of novel therapies and vaccine strategies.

### Type-I IFNs

The group of type-I IFNs includes 13 highly homologous IFN-α genes, as well as single genes encoding IFN-β, -ε, -κ and -ω. All type-I IFNs are encoded on chromosome 9^2^ and almost all cell types can elicit a type-I IFN response. Binding of any of the type-I IFNs to a receptor subunit leads to the corecruitment of a second subunit and dimerization of the IFN-α/β receptor complex (IFNAR).^12^ The receptor subunits are associated with Janus-activated kinases (JAK1) and tyrosine kinase 2. Their interaction leads to the phosphorylation of signal transducer and activator of transcription (STAT)-1 and -2. In specific cell types, STAT-3, -4, -5 and -6 can also be phosphorylated.^13,14,15^ STAT-1/2 heterodimer interactions initiate the corecruitment of INF regulatory factor (IRF)-9 and consequent binding to the intra-nuclear IFN-stimulated response element. In contrast, a STAT-1 homodimer binds to the IFN-γ activated site enhancer elements in promoters of dozens of IFN-stimulated genes (ISG) and induces transcription.^15,16,17^

### Type-II IFNs

IFN-γ is the sole representative of type-II IFNs. IFN-γ is not induced via direct sensing of virus fragments but rather through stimulation of epitope-specific T cells and natural killer cells. Similar to IFNAR, the receptor for IFN-γ (IFNGR) is a heterodimer of IFNGR1 and IFNGR2.^18^ The receptor subunits are associated with JAK1 and JAK2 tyrosine kinases. The interaction induces phosphorylation of STAT-1. IFNGRs are broadly expressed on many cells and induce anti-viral ISGs.^18,19^

### Type-III IFNs

The IFNL family is the most recently discovered group of IFNs, comprising four homologous members: IFNL1 (interleukin (IL)-29), IFNL2 (IL-28A), IFNL3 (IL-28B)^9,10^ and the very recently described IFNL4.^20,21^ All are encoded on chromosome 19 (19q13.13 region). Formally, IFNLs belong to the IL-10 family of cytokines containing IL-10, IL-19, IL-20, IL-22, IL-24 and IL-26. The IFNL coding region has five exons and four introns. Their positions in relation to the open reading frames are conserved for all IFNL and IL-10-related cytokines.^22,23,24^

**Figure 1:**
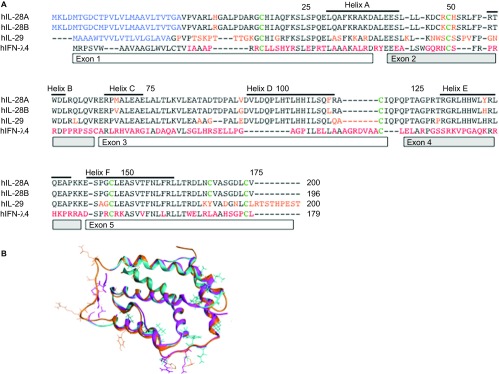
(**A**) Sequence alignment of IFNLs. An amino-acid sequence alignment of human IFNL1–4 is shown. Red letters indicate significant differences. Green letters indicate common cysteines. Helices and exons are indicated with boxes. (**B**) Sequence alignment overlay for different IFNLs. Amino acids of significance are highlighted.

The high degree of amino-acid sequence similarities within IFNL1–3 suggests a common ancestor gene.^21^ IFNL2 and 3 share 96% sequence similarity, whereas IFNL1 is less similar and also differs in terms of the pattern of disulfide bridges.^25^ IFNL4 arises as a consequence of a frameshift mutation generating a new gene not normally expressed, and demonstrates only a 40.8% similarity to IFNL3. Figure 1A shows a sequence alignment of all IFNLs. Figure 1B highlights the differences in side chains between IFNL1–3 in three-dimensional structures. Despite almost identical sequences, IFNL3 and IFNL2,^9,26,27^ have markedly different anti-viral activities. IFNL3 demonstrates the highest anti-viral activity as measured by HepG2 challenge with encephalomyocarditis virus. IFNL3 had a 16-fold lower half-maximal effective concentration (EC_50_) compared to IFNL2 and two-fold lower EC_50_ value relative to IFNL1.^28^ Additionally ISG induction (Mx1 and IRF9) by IFNL3 was significantly higher compared to IFNL1 and IFNL2.^29^

## EXPRESSION OF IFNLS

Almost any cell type is able to express IFNL1–3 mRNA in response to diverse viral infections such as Sindbis virus, dengue virus, vesicular stomatitis virus, encephalomyocarditis virus,^9,10^ respiratory syncytial virus,^30,31^ influenza virus, Sendai virus^32,33^and hepatitis C virus (HCV).^34,35,36^ High levels of IFNLs, but not IFN-α, were observed during viral infection of lung and liver tissues,^36,37,38,39,40,41^and IFNLs seem to be the major IFNs induced in airway epithelial cells during infection with respiratory viruses.^42,43,44^ The most potent producers of IFNLs however, seem to be myeloid and plasmacytoid dendritic cells (DCs).^27,45,46,47,48^

The expression kinetics of IFNL1–3 may be cell type dependent and have not been fully elucidated. However, our data suggests a clear difference in the expression kinetics between different IFNL family members during stimulation of fibroblasts and human peripheral blood mononuclear cells (PBMCs). IFNL3 showed a peak at 24-h after stimulation with cytomegalovirus (CMV), whereas IFNL1 peaked already at 6 h when measuring mRNA levels. Stimulation with poly(I:C) showed a significantly earlier peak of IFNL expression compared to infection with CMV.^49^

**Figure 2:**
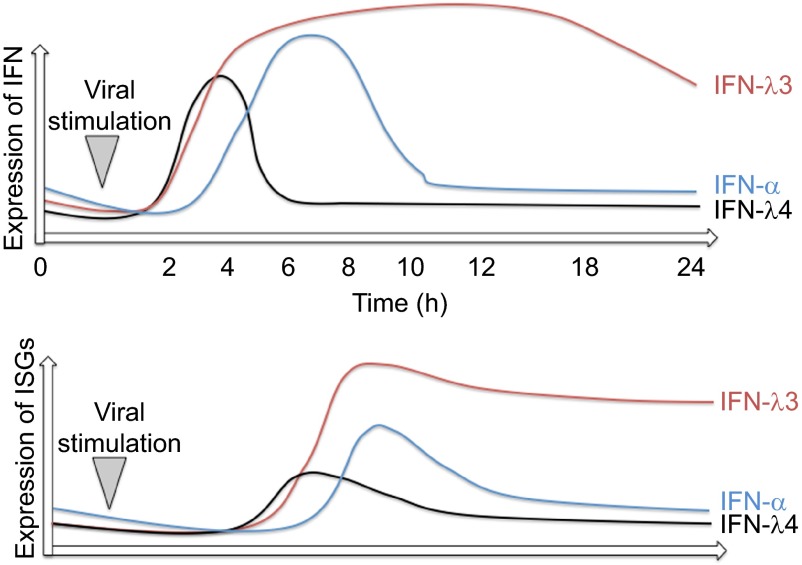
Expression dynamics of IFNs and ISGs during viral infection or inflammatory stimulation. Red line represents IFNL3, blue line IFN-α and black line IFNL4 expression dynamics. The lower panel illustrates the amount of ISG expression.

IFNL4 mRNA could be detected in primary human hepatocytes (with the required single nucleotide polymorphism (SNP)) 2–4 h after stimulation with poly(I:C) and after *in vitro* infection with HCV. However, a rapid downregulation of IFNL4 mRNA expression at 8 h was observed. In contrast, IFNL3 mRNA was still expressed 24 h after stimulation (Figure 2).^21^ The rapid downregulation after stimulation may suggest either a lack of an amplifying positive feedback loop, or rapid induction of a specific negative feedback mechanism. Interestingly, in liver biopsies from non-HCV-infected patients, a baseline mRNA expression of IFNL4 could be observed.^21^ Although this may suggest that IFNL4 is expressed in conditions unrelated to HCV, activation of this signaling pathway may happen through a wide spectrum of stimuli. DCs have not yet been specifically explored as a source of IFNL4.

Purported explanations for the variance in expression kinetics and subsequent ISG expression (see below) may include: transcription factor differences in specific cell types; the presence of SNPs regulating IFNL expression (cell types have rarely been genotyped to determine the background of the original cell); regulatory feedback loops; receptor expression, and receptor binding affinity. In addition, various viruses may have evolved specific defence mechanisms based on the cytokine and IFN microenvironment which ultimately determines the viral tropism.^50^ The IFN-α signaling cascade is known to be modulated at numerous points^51^ and it is also very likely that co-evolutionary competition between IFNLs and different viruses has occurred. Of note, the replication of emerging respiratory viruses such as influenza H7N9 or Middle East respiratory syndrome coronavirus may be substantially controlled by IFNLs. Many ‘new' viruses have shown an ability to interfere with the IFN signaling pathways or involved antiviral ISGs.^51,52,53,54^ Multiple factors likely act to influence IFNL-associated immune responses and subsequently impact outcomes of infectious diseases.

### Transcription factors

Both, type-I and -III IFNs are directly induced through sensing of viral infection via pattern recognition receptors such as Toll-like receptors (TLRs), retinoic acid-inducible gene 1 or melanoma differentiation-associated protein 5.^17,51^ For example: CpG-DNA (TLR-9 agonist) induces the expression of IFN-α, -β and IFNL1-3 in plasmacytoid DCs.^27,33,46,47,55^ Lipopolysaccharide (TLR-4 agonist) and poly(I:C) (TLR-3 agonist) induce IFN-β and IFNL1–3, but not IFN-α in myeloid DCs.^33^ Poly(I:C) can also induce IFNL4 expression in the appropriate genetic background.

The co-expression of type-I and -III IFNs is explained by common transcription factors and binding sites in the promoter regions. Specifically, binding sites for activator protein 1, IRF3, IRF7 and NF-κB were predicted in the promoter region of all IFNL genes.^32,33,56,57,58,59,60,61,62^ Further, the promoter regions of IFNL1-3 share very similar or the same transcriptional regulatory elements. Recently, another important transcription factor of IFNLs, Med23, was discovered.^63^ Med23 significantly upregulated the expression of IFNL mRNA and protein level by directly interacting with the transcription factor IRF7. The synergistic effect of Med23 and IRF7 on IFNL induction suggests that this may be a particularly important transcription factor. Transcription factor binding sites for IFNL4 have not yet been characterized in detail.

### Single nucleotide polymorphisms

A series of SNPs near the IFNL3 and IFNL4 genes have been discovered and importantly are noted to be in high linkage disequilibrium.^26,64^ Although the SNPs are widely distributed and some are located considerable distances from coding sequences, they potentially impact the binding of transcription factors and methylation sites,^65,66,67,68^ thereby influencing the promoter output. The rs4803217 SNP in the 3′ untranslated region of the *IFNL3* gene has been associated with mRNA stability.^69,70^ Some of these SNPs are possibly just in linkage with other functional SNPs (e.g., rs12979860 with ss469415590). Thus, deciphering the relative clinical importance of an individual SNP is rendered very difficult. Table 1 summarizes the locations and impact of published SNPs involving the IFNL signaling cascade.

The impact of the SNPs on IFNL expression is debated. Most studies have concluded that the SNPs rs12979860 and rs8099917 are associated with reduced IFNL3 expression during chronic HCV infection in liver biopsies,^71,72,73,74^ serum,^75^ and PBMCs.^49^ In addition, in CMV-infected fibroblasts and stimulated PBMCs, SNPs in rs12979860 were associated with lower IFNL3 gene expression.^49^ To the best of our knowledge, no study has shown a significant increase in IFNL3 expression due to a SNP. There is a high likelihood that SNPs may also affect the function of other genes in the locus. These variants have been associated with alteration in serum IFNL1 and IFNL2 levels and with HCV infection outcomes, both spontaneous resolution and IFN treatment responses.^34,74,76^

The impact of the ss469415590 SNP on the expression of IFNL4 is well defined. In the context of a delta-G polymorphism, the frameshift mutation generates a ‘new' gene.^21^ Based on the genotype distributions of the strongly linked rs12979860 SNP, we could assume that almost 40% of Caucasians express IFNL4.

### IFN-lambda receptor expression

The IL-28R consists of two subunits. IFNL first binds to the IL-28RA (α-subunit; encoded on chromosome 1), followed by the corecruitment of the IL-10RB (β-subunit; encoded on chromosome 21).^25,77,78,79,80^ In a similar fashion to the IFNAR receptor–ligand interaction, the dimerization of the receptor leads to the activation of JAK1 and tyrosine kinase 2 and phosphorylation of STAT-1 and -2. STAT1, STAT2 and IRF9 combine to form the IFN-stimulated gene factor-3 transcription factor complex, which induces the expression of multiple ISGs.

IL-10RB shows a broad expression pattern,^81^ whereas in contrast, the IL-28RA subunit is more restricted. A high expression of IL-28RA mRNA could be found in lung, intestine, and liver tissues with epithelial cells considered to be the main responders to IFNL exposure. A few other immune cells express IL-28RA especially at the mRNA level (e.g., B cells, macrophages and plasmacytoid DCs), but conflicting protein expression data are reported in the literature. Expression in brain tissue was low.^9,10,45,82,83,84,85^ IL-28RA also exists as a membrane bound form and a secreted form lacking exon VI, representing different splice variants.^10,82,86^ The secreted receptor may bind and inhibit IFNLs.

### Regulation of receptor expression

Important transcription factor binding sites have been identified within the promoter region of the *IL-28RA* gene (e.g., STAT1, AP-2 and p53).^87^ This points to an upregulation of IL-28RA gene expression during stimulation with various IFNs. During maturation of monocytes to macrophages a significant upregulation of IL-28RA surface expression could be observed.^88^ The expression of IL-28RA on hepatocytes seems to be different between cell lines such as Huh7 or HepG2 (responsive to IFNL) and primary hepatocytes.^89,90,91,92^ Primary hepatocytes show an initial low responsiveness to IFNL due to a low baseline mRNA expression of IL-28RA, however during treatment with IFN-α a marked upregulation of IL-28RA mRNA was observed.^88,89^ Interestingly this upregulation was significantly enhanced in patients with a minor allele SNP genotype (rs12979860 and rs80999917) in IFNL3. This suggests the existence of a potent regulatory feedback loop between IFNL and IL-28RA.^89^ Although somewhat speculative at the current point, this increase in IL-28RA mRNA may be mediated by IFNL4 expression in the appropriate genetic background. During CMV infection of fibroblasts, the mRNA of IL-28RA increased by about two-fold, but protein expression levels (western blots) remained stable.^49^ Though requiring further definitive elucidation, these findings support considerable interplay in initial IFN mediated anti-viral responses, the ultimate outcomes of which are heavily influenced by the genetic background of the host.

Like IFNAR, IL-28RA could be modulated during infection by viral proteins or host and viral microRNAs.^93^ Interestingly, multiple SNPs in IL-28RA have been described (rs10903032, rs10903034, rs10903035, rs11249002 and rs11249006). The rs10903035 SNP (GA or GG) is within the 3′ untranslated region of the IL-28RA mRNA sequence and was an independent risk factor for IFN-α treatment failure against HCV (odds ratio: 2.0; 95% confidence interval: 1.19–3.36).^46,94^ This SNP has a relatively high minor allele frequency of 41.1% (ref SNP NCBI database). According to the 1000genome tool of the NCBI, the risk allele ranges from 25% (Iberian population in Spain) to 55% (Southern Han Chinese). The mechanism of how the SNPs in the *IL-28RA* gene affect IFN-α treatment outcomes has not been studied.

Interestingly, the rs10903035 IL-28RA SNP has been associated with insulin resistance in HIV/HCV co-infected patients.^95^ Another, rs4649203 has been linked to a risk of psoriasis in four independent populations,^96^ and to a risk to develop systemic lupus erythematosus.^97^ Although the IFNL3 SNPs have not been associated with treatment response of IFN-β in multiple sclerosis,^98^ the IL-28RA SNP itself was associated with an increased risk for multiple sclerosis.^99,100^ These observations suggest important influences on adaptive immune cells, which are tightly connected to autoimmune disease.

### The ligand–receptor complex

The ligand interface includes helix A, loop AB and helix F (Figure 1). The IL-28RA interface arises from the N-terminal domain and interdomain hinge region. The extracellular region of IL-28RA has two β-sandwich domains D1 and D2. The interaction region consists largely of van der Waals and hydrophobicity.^25^ The most important amino acids on the ligand for interaction with IL28RA are located in the AB loop: Lys49 and Arg51 in IFNL3 and Arg49 and His51 in IFNL2, respectively.^11^ Binding to IL-10RB is important via the helix D amino acids: Gly95 in IFNL3 and Val95 in IFNL2. Of note IFNL4 differs in the area corresponding to the D helix of IFNL3.^21^ Various mutations in the receptor binding sites of IFNL3 and IFNL1 have been explored in terms of functionality ^11,25,77^.

The observed disparities in inducible responses to IFNs may arise due to relative differences in IFNL subtype expression, different ligand binding affinities and competition among IFNs for the same receptor. Rather than the affinity to the individual subunits, the stability of the ternary interferon receptor complex might be central to explaining the differences between the IFNL cytokines, akin to that observed with IFN-α.^101^ To date, no complete model for the ligand–IL-28RA–IL-10RB complex exists.

## MAJOR FUNCTION OF IFNLS: DIRECT ANTI-VIRAL CONTROL

Not surprisingly, IFNLs share many biological activities with IFN-α, in particular direct anti-viral effects.^15,102,103^ Differences with reference to IFN-γ may be explained by the expression dynamics of IFN-γ, which is only secreted by a subset of immune cells such as natural killer cells and pathogen-specific T cells. In addition, the IFNGR expression differs and therefore, these effects are not comparable to IFN-α and -λ.^104,105,106^

IFN-α-treated Huh7 cells demonstrate a short induction of STAT1 phosphorylation (30 min–4 h), followed by a sharp reduction in ISG expression, temporally associated with the induction of the anti-inflammatory ISG USP18 (ubiquitin carboxy-terminal hydrolase 18). IFNL on the other hand induces both a later and more sustained phosphorylation of STAT-1 over 24 h^29,107^ (more details discussed below). The antiviral activity of IFNLs has been demonstrated in cell cultures infected with murine CMV,^83^ HIV,^40^ influenza virus,^108,109^ hepatitis B virus^91^ and HCV.^84,91,110^ In addition, their role in direct antiviral effects *in vivo* has also been demonstrated in IL-28RA and STAT1 knockout animals, where a significant increase in viral replication was observed.^108,109,111^

As mentioned previously, binding of IFN type-I and -III to their respective receptors induce a variety of ISGs.^15^ Anti-viral ISGs interfere with viral replication at different stages.^15,17^ Important examples are ISG15, MX1, OAS1-3, and PKR (proteins of 15 kDa; the dynamin protein family of Mx GTP-ases; 2′5′-oligoadenylate synthetase; and protein kinase R, respectively). In contrast, the antiviral signaling is controlled by anti-inflammatory ISGs including USP18 and SOCS1-3 (suppressor of cytokine signaling), which interfere with the STAT signaling cascade. They function as part of a negative feedback loop to limit the extent and duration of the IFN response.^112,113^ This feedback mechanism seems to be inherently different for IFN-α and IFNLs (see details below).

Limited biological activity attributable to IFNL4 is only now emerging following its original description. IFNL4 can induce ISG expression in HepG2 cells and anti-viral effects are also described against HCV infected Huh7-lunet cells, as well as for coronaviruses in human airway epithelial cell culture.^20^ Blockade of IL-10RB with siRNA and blocking antibodies significantly reduced the activity of IFNL4. Compared to IFNL3, however, IFNL4 demonstrated an almost 10-fold lower EC50 in a human embryonic kidney (HEK293) cell luciferase reporter system.^20^ The efficacy of his-tagged IFNL4 secretion seems to be significantly lower compared to IFNL3, which may account for the lower EC50.^20,21^

### Differences in ISG expression between IFN types

A high overlap of gene induction between type-I and -III IFNs exists. However, in hepatocytes treated with IFN-α and IFNL3, differential effects on ISG expression levels and temporal kinetics have been described.^114,115,116^ IFN-α stimulates a rapid peaked induction of ISGs, whereas IFNL3 induces a slower, but more sustained increase in gene expression (Figure 2). IFN-α induced genes are generally associated with leukocyte chemotaxis, IFN signaling and lymphocyte proliferation, whereas IFNL3-induced genes are associated with antigen presentation and IFN signaling.^114^ More specifically, 6 h after stimulation with IFN-α, 134 genes were upregulated, with only 13 genes after 24 h. In contrast, 6 h after stimulation with IFNL3, 40 genes were upregulated, with 580 genes upregulated after 24 h. Of particular note is the differential expression profile of certain immunomodulatory genes. USP18 and SOCS1 were downregulated over 24 h in IFN-α-treated hepatocytes (USP18: 16.5- to 4.2-fold; SOCS1: 2.3- to 0.5-fold). IFNL3 on the other hand upregulated USP18 and SOCS1 over 24 h (USP 18: 13.6- to 19.6-fold; SOCS1: 3.3- to 6.8-fold).^114^ USP18 was shown to be necessary and sufficient to induce differential desensitization by impairing JAK1 at the IFNAR.^117,118,119^ Although these expression profiles may be cell type-specific, the potent and sustained effects of USP18 upregulation in the context of a chronic infection may significantly affect IFN-α induced signaling.

RNA sequencing of HepG2 cells transfected with IFNL4 and treated with IFN-α and IFNL3 suggests that some genes known as markers of HCV-induced liver damage, such as *rantes* and *fos*, were specifically induced by IFNL4 but not by other IFNs. This suggests specific functional roles for IFNL4, distinct from IFN-α and IFNL3.^21^ During infection with HCV, the expression pattern of many ISGs significantly change, most likely due to immunomodulatory effects of HCV proteins and complex inhibitory effects of both IFN signaling pathways.^115^

## IMPACT OF IFNL SNPS ON INNATE ANTI-VIRAL EFFECTS OF IFNL3 AND IFNL4

The role of IFNLs in the host response to virus has been most well studied in HCV infection. HCV infection has been classically treated with (pegylated) IFN-α-based therapy to stimulate a robust antiviral immune response. Baseline ISG expression is considered an important marker of successful IFN-α treatment of HCV.^72,120^ Interestingly, patients with chronic HCV infection and non-responsiveness to IFN-α treatment show a higher baseline hepatocyte ISG expression.^121,122,123,124,125,126^ Elevated basal ISG expression and treatment failure have both been linked to SNPs in the *IFNL3/4* and *IL-28RA* genes. The clinical importance of these SNPs is best explored in HCV infection and discussed in detail below. Importantly, however, the role of SNPs in the IFNL signaling may also be highly important for existing ‘old' and ‘emerging' pathogens. Though beyond the scope of this review, it is also conceivable that such polymorphisms shaped the tropism of viruses and influenced viral evolution and will continue to do so.^50,127^

### IFN-feedback mechanism

Insight into the higher baseline ISG expression was provided through genome wide association studies of patients with chronic HCV infection. SNPs in the IFNL3 gene were associated with a risk of progression to chronic infection and significantly lower rates of HCV clearance in response to IFN-α-based treatment.^26,74,76,128^ Many SNPs (in particular rs12979860 and rs8099917) in the IFNL3 gene region were associated with lower IFNL3 expression levels and higher baseline ISG expression,^71,110^ suggesting the existence of a regulatory feedback mechanism. IFNL treatment and expression in hepatocytes is associated with potent induction of ‘immunoregulatory' ISGs, such as USP18 and SOCS1, whose duration of expression is comparatively longer compared to IFN-α.^29,107,114^ These ISGs act in an inhibitory capacity to dampen pro-inflammatory cascades. Likewise, in liver biopsies from HCV-infected patients with a major allele genotype, A20 and ZC3H12A were upregulated.^123^ A20 is a key inhibitor of NFκB signaling. ZC3H12A destabilizes the mRNA of IL-6 and IL-12p40—both potent inducers of an inflammatory response.^129^ The enhanced expression of these immunoregulatory ISGs may lead to a cumulative reduction of anti-viral ISG expression, thereby modulating the innate immune response to viruses.^121,122,123,124,130,131^

The paradoxical situation of higher ISG expression in IFNL3 SNPs despite lower *IFNL3* gene expression could be explained by (i) a disruption in the temporal regulatory aspects of type-I and -III IFN-signaling cascades (at the level of either ligand or receptor); or (ii) the actual expression of IFNL4 in the context of reduced IFNL3 by virtue of high IFNL SNP linkage. Based on the current knowledge and our work, we favor the former, as IFNL4 secretion seems to be low and the biological impact in comparison to other IFNL proteins would seem less potent. We postulate, that IFN-α induces a more potent but temporally limited induction of ISGs due to a negative feedback loop (via SOCS1 and USP18, see above). In contrast, IFNLs, and in particular IFNL3 induce a somewhat ‘weaker' but more sustained ISG expression comprising both anti-viral and immunoregulatory profiles (Figure 2).

**Figure 3:**
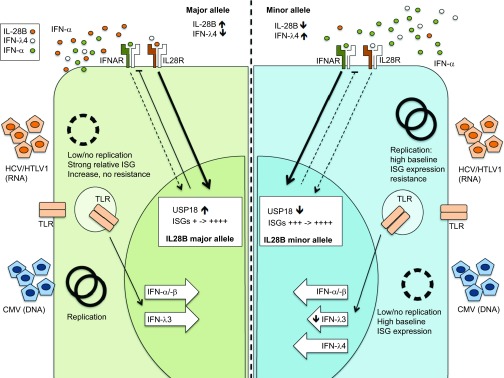
Hypothesis of IFNL3 single nucleotide polymorphism and impact on virus replication. The left panel shows a major allele genotype with a presumed fully functional IFNL3 expression during virus replication. The right shows a minor allele genotype with reduced IFNL3 expression, but increased IFNL4 expression. The genotype results in a significantly different ISG background expression which may be an advantage or disadvantage for different viruses.

Figure 3 illustrates a proposed hypothesis regarding the feedback mechanism between IFN-α and IFNL3/4 according to specific genotypic backgrounds and in response to differing infection scenarios; acute and chronic viral infection (Figure 3). During acute viral infection, the expression of both IFN-α and IFNLs is triggered. In the context of a major allele IFNL3 genotype, IFNL3 is highly expressed and may better inhibit the initial replication of a virus due to a longer lasting anti-viral effect of induced ISGs. This may culminate in virological control and ultimately affect spontaneous clearance of virus. Clearly such outcomes will be reliant on efficient virus-specific T-cell priming, where a key role for IFNL is also proposed.

In contrast, in the setting of a minor allele IFNL3 SNP, the amount of IFNL3, and subsequent anti-viral and modulatory ISG induction, is significantly lower. Thus, a sharp but limited IFN-α response predominates with high, short-lived ISG expression. This can foster breakthrough replication of viruses and subsequent selection of quasi-species resistant to ISGs (especially relevant to RNA viruses lacking polymerase proof reading capacity such as HCV). The constant viral replication in conjunction with impaired IFNL3 mediated regulatory activity amplifies IFN-α-mediated ISG expression further. In principle, the anti-viral properties of ISGs are similar to those of antiviral therapy where the threshold of replication is determined by a minimal inhibitory concentration.^132,133,134,135^ If the exposure of the virus towards ISGs is on occasion, as a consequence ‘subtherapeutic' mutation can occur with selection of quasi-species.

Aside from this proposed regulatory interplay between IFNs, the actual expression of a new player, IFNL4, could itself provide explanation for the elevated baseline ISG expression. It was recently shown that the expression of IFNL4 directly correlated with IFI44, IFIT1 and MX1 expression in hepatocytes.^136^ It remains to be seen if indeed the activity of this ‘new' gene product can directly account for the differing phenotypes observed.

At present, we would propose that the higher baseline expression of ISGs in patients with a minor allele genotype arises due to a combination of persistent activation of the IFN-α signaling cascade and a general reduction of inhibitory signals such as USP18 due to relative reductions in IFNL3 expression. In addition, it is likely that the newly discovered SNP in IL-28RA stands to affect all IFNL signaling, further disrupting the IFN-α and IFNL cross-talk. The clinical implications of this model are discussed in the next chapter.

### Clinical impact of SNPs

The impact of SNPs in IFNL3 has clearly been defined for HCV infection.^26,74,76,128^ Minor allele carriage has been associated with high ISG background, and selection of quasi-species of ISG resistant HCV genomes^131^ (see above suggested model). Furthermore in this context exogenous IFN-α is incapable of effecting sufficient ISG induction from the relatively high baseline level that exists. Using the model of liver transplantation in HCV illustrates some of these aspects. Recently, it was shown that liver transplant recipients demonstrate an initial lower hepatitis C viral load if the donor liver graft has a minor allele genotype, and the recipient has a major allele genotype.^137,138^ In this specific constellation, a relatively ISG-susceptible virus (from the major allele recipient) has to replicate in a high ISG expression background (minor allele donor graft), which may result in improved initial control of replication. With iatrogenic immunosuppression, the adaptive immune response is impaired meaning viral replication control is achieved preferentially by the innate immune response. The reduced expression of IFNL3 (minor allele donor graft) allows a more potent IFN-α response with earlier control of the virus; however, with chronicity selection for resistant species ultimately occurs.

The role of various SNPs in the IFNL signaling cascade for other viruses is not as well established.^139^ In particular for the human T-lymphotropic virus 1, the impact of the minor allele genotype for IFNL3 (rs12979860) is unresolved following conflicting reports in terms of disease progression.^140,141^

Considering the above, it could be proposed that DNA viruses with accurate proof reading such as cytomegalovirus (during acute virus infection) would be at a disadvantage in a high ISG environment, as the development of escape mutants may take too long. Indeed, solid organ transplant recipients at high risk for primary (acute) CMV infection (donor sero-positive, recipient sero-negative) with rs8099917 minor allele genotype were in fact at decreased risk for CMV viremia in the first year post-transplant.^49^ A similar observation was made in hematopoietic stem cell transplant recipients where a trend for reduced risk of CMV replication and lower CMV viral loads was observed in a minor allele background.^142^ One potential explanation for this includes the combination of a higher anti-viral ISG background in the context of lower immunoregulatory activity. Without a doubt adaptive T-cell responses also make a telling contribution, which we will address in the following paragraphs.

## EXPANDING ROLE FOR IFNL: FROM INNATE TO T- AND B-CELL MODULATION

### Macrophages

Macrophages show marked phenotypic heterogeneity.^143^ Functional diversity results from a differentiation programme that is subject to environmental imprinting. Macrophages can regulate three different homeostatic activities—host defence, wound healing and immune regulation. Although a range of macrophages have been described, classically two types of macrophages can be differentiated: M1 and M2. The M1 phenotype is differentiated from the M0 phenotype in response to IFN-γ.^144^ M1 cells produce high amounts of IL-12 and IL-23, but low amounts of IL-10. In addition, effector molecules such as reactive oxygen species and inflammatory cytokines such as IL-1, tumor-necrosis factor-α and IL-6 are produced. A key feature is the induction of Th1 cells. M2 cells in contrast, are induced by IL-4 or IL-13. M2 cells do not present antigen to T cells and produce minimal amounts of pro-inflammatory cytokines. A key feature is the induction of Th2 cytokines and subsequent stimulation of T regulatory cells.^145,146^ Macrophages thus are positioned at the intersecting innate and adaptive immune responses continuously modulating the Th1 and Th2 cytokine balance during infection.

**Figure 4:**
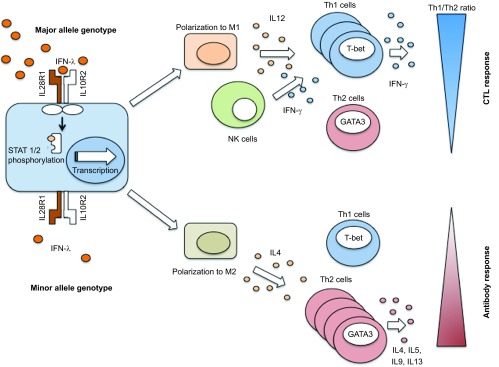
Impact of IFNL on Th1/Th2 cytokine profiling. The IFNL3 major allele genotype shows a predominant polarization to a macrophage M1 phenotype, whereas the minor allele genotype may show a relative increase of M2 phenotype. These macrophage phenotypes are associated with the downstream regulation and polarization of Th1 and Th2 cells. NK cells, natural killer cells.

Interestingly, the differentiation cascade of monocytes to macrophages is influenced by IFNL.^88^ IFNL1 increases TLR-induced IL-12p40 production in human monocyte-derived macrophages. Furthermore, IFNL1-treated macrophages were more responsive to IFN-γ, because IFNL1 enhanced IFN-γ-induced IL-12p40 and tumor necrosis factor production by macrophages following R848 stimulation. In addition, IFNL1 upregulated, whereas IFN-α downregulated, the surface expression of the IFN-γ receptor α-subunit on macrophages, which affected the responsiveness of TLR-challenged macrophages to IFN-γ.^88^ This data highlights the diverse effects of IFN-α and IFNL1 in modulating macrophage populations. The influence of IFNL3/4 SNPs and IL-28RA genotypic background on macrophage polarization and function remains to be studied, but affecting macrophages in general will have a significant impact on host responses to infection (Figure 4).

### Dendritic cells

Similar to macrophages, DCs function at the interface between innate and adaptive immune responses. In mice, CD8α^+^ DC promote T-cell skewing towards a Th1 lineage through the secretion of IL-12, while the CD8α^−^ DC subsets were linked to Th2 differentiation.^147^ Human BDCA-3^+^ DC share several relevant characteristics with CD8α^+^ DC. The expression of IL-28RA on human DCs has been mostly seen on plasmacytoid DCs,^46^ while conflicting reports exist for expression of IL28RA on human monocyte derived DCs, possibly due to whether one looks at protein or mRNA.^88,148^ To the best of our knowledge, however, no functional differences have been studied in the context of differing IFNL genetic backgrounds.

### Th1/Th2 balance

Virus-specific Th1 and cytotoxic T cells are crucial to control viral replication, whereas B cells receive important growth signals from Th2 cytokines.

Antigen-presenting cells such as DCs and macrophages play a decisive role in the differentiation of T-cell subsets into a Th1 or Th2 phenotype. IL-12 and IFN-γ are the critical cytokines initiating the downstream signaling cascade for the differentiation of Th1 cells.^149^ IL-12 is secreted in large amounts by antigen-presenting cells, in particular DCs and macrophages, following their activation through pathogen detection via pattern recognition receptors.^150,151^ IL-12, in turn, induces natural killer cells to produce IFN-γ. The major transcription factor to induce Th1 cells is T-bet.

IL-4 and IL-2 are critical for Th2 differentiation. The major transcription factor involved in Th2 lineage differentiation includes the IL-4-induced STAT6, which upregulates the expression of the master regulator GATA3.^152,153,154,155^ The expression of T-bet and GATA3 have opposing effects.^156^

Treatment of PBMCs with IFNLs have been shown to increase Th1 and suppress Th2 cytokine producing T cells.^157,158,159,160,161^ The IL-28R surface expression by immune cell fractions remains somewhat controversial. Though some authors have demonstrated mRNA expression of IL-28RA in T cells, this does not necessarily correlate with functional surface expression. In our hands, T cells do not express a surface IL-28RA protein (unpublished data). However, others have reported that purified naive and memory human CD4 T cells express IL-28RA and that these cells were responsive to IFNL1. The expression of Th2 cytokines (IL-4 and IL-13) was suppressed in CD4 T cells by IFNL1. Further GATA3 expression was reduced by IFNL1 treatment,^157^ and super-antigen stimulated cells pre-treated with IFNL1 showed significantly lower levels of IL-13. Strikingly, the IL-13 secretion was 100-fold more sensitive to IFNL1 treatment compared to IFN-γ. Interestingly, naive T cells exposed to allogeneic myeloid DC that had been matured in the presence of IFNL1, demonstrated a similar reduction in IL-13 production, IFN-γ secretion being unaffected.^158^ Taken together, this suggests that myeloid DC function may be significantly altered by IFNLs and have important downstream effects on the Th1/Th2 balance.

Likewise, IFNL3 treatment of lung DCs in a mouse model of asthma significantly increased Th1 cytokine and reduced Th2 cytokine expression.^159^ This activity may be of particular importance for immune-mediated lung diseases such as asthma. Indeed, in children with asthma, it was observed that those patients with a minor allele genotype had lower IFNL3 levels in BAL samples, but also significantly higher eosinophil granulocytes in sputa samples with higher Th2 cytokines.^162,163,164,165,166^

Keeping to the respiratory microenvironment blockade of the IL-28R markedly reduced the RSV-mediated suppression of CD4 T-cell proliferation, suggesting a specific role for IFNL signaling.^167^ We too observed a similar phenomenon with naïve CMV-specific T cells from sero-negative healthy blood donors demonstrating a reduced proliferation capacity when pre-treated with IFNL3 and stimulated with CMV lysate.^49^

The modulation of IFNL expression by SNPs in the IFNL3/4 gene region and IL28RA signaling cascade may potently affect Th1 and Th2 cytokine production and memory formation during viral infections. This remains to be fully explored but clearly represents a modulation of a critical component of the host response to pathogens.

### B-cell activation

Th2 cytokines are known to promote optimal B-cell activation. Therefore, modulation of Th2 cytokines by IFNLs can profoundly affect B-cell functions (proliferation and antibody production). One study has examined the effects of IFNL1 and IFNL2 on the activation of B cells by anti-CD40 with/without cytokines. Treatment with IFNL1 or IFNL2 was able to modestly downregulate only IgG4 production in response to both anti-CD40 and IL-4.^168^

We would postulate that modulation of IFNL by virtue of a skew away from Th1 to Th2 could result in a propensity towards increased B-cell activation states in response to stimulation. Indeed, in a large cohort of healthy children vaccinated against measles, children with a minor allele genotype (rs10853727) had significantly higher post-vaccine antibody titers (median 807 mIU/mL versus 1727 mIU/mL).^169^ This actually suggests that the SNP potentially affecting IFNL4 expression may have a significant impact on antibody production in general; against pathogens, in response to vaccination, or in autoimmune diseases. This important association has to be explored in future studies.

## SUMMARY

Infectious diseases are a perpetual threat with novel emerging entities a continuous challenge. Despite enormous advances in the understanding of the immunological processes involved in responses to pathogens, viral infections remain a theme of considerable unmet need. The discovery of IFNLs, and even more the determination of common SNPs, which affect their expression and biological function, has opened a new chapter and illustrates the intricate immunomodulatory activity, which critically determines infection outcomes. IFNL has direct antiviral functions, common to other IFNs; however, complex regulatory mechanisms exist which have implications on viral infection. Intriguingly, IFNL demonstrates activity at the interface of innate and adaptive immunity. Dynamic immune responses differ according to host IFNL3 genotypes with varied consequences for distinct viruses both those in existence and those now emerging. New data continue to emerge placing IFNL at the core of host responses effectively coordinating the fine-tuned activation of innate and adaptive immune responses. The long-term effects on the Th1/Th2 balance might have implications for the priming of T- and B-cell memory responses, but also for vaccine responses and autoimmune disease prevalence. Manipulation of IFNL signaling provides a means to specifically tailor and individualise therapeutic manipulations in favor of the host perhaps even taking into account the nature of the infecting pathogen. Future work will conclusively determine the role of IFNL SNPs in diverse viral infections outside of HCV. This signaling cascade appears optimally positioned to orchestrate or be manipulated to effect robust and durable responses to an ever-increasing array and complexity of pathogens.

## Figures and Tables

**Table 1:** Selection of important SNPs in the IFNL signaling cascade

Gene	SNP	Nucleotides	Localization	Impact on expression (minor allele)	Impact on virus replication (minor allele)	Impact on adaptive immune functions (minor allele)	Reference
IFNL4^a^ (IFNL3)	rs12979860	Major: C/C Minor: C/T, T/T	—	Reducing IFNL3	HCV: reduced IFN-α treatment response, lower rates of spontaneous clearance		49, 71–75
					Acute CMV: reduced replication		
None^b^	rs8099917	Major: T/T Minor: T/G, G/G	—	Reducing IFNL3	HCV: reduced IFN-α treatment response, lower rates of spontaneous clearance	Acute CMV: less priming of CMV-specific T cells	71–74
					Acute CMV: reduced replication		
IFNL4^a^	rs10853727	Major: A/A		?	-	Higher post-vaccine measles titers	76
(IFNL3)		Minor: A/G, G/G					
IFNL4	ss469415590	Major: T/T	CDR	Frameshift, causing IFNL4 expression	HCV: reduced IFN-α treatment response, lower rates of spontaneous clearance	?	20, 21
	(rs368234815)	Minor: T/ΔG, ΔG/ΔG					
IFNLR1	rs10903035	Major: A/A Minor: G/A, G/G	3′ UTR	?	HCV: reduced IFN-α treatment response, lower rates of spontaneous clearance	?	77

a Originally some SNPs were assigned to or close to IFNL3; however, with the discovery of IFNL4,^21^ there are various changes. We show both locations, although the rs129 79860 and the rs10853727 have their ‘true' location in IFNL4.

b Originally the SNP rs8099917 has been assigned to IFNL3; however, its true location is between IFNL4 and IFNL4P1.

?, not known.
